# Fatigue Life Analysis of a Plate with a Repair Node Subjected to Uniform Shear

**DOI:** 10.3390/ma19030604

**Published:** 2026-02-04

**Authors:** Iga Barca, Marek Rośkowicz

**Affiliations:** Faculty of Mechatronics, Armament and Aerospace, Military University of Technology, 00-908 Warsaw, Poland; marek.roskowicz@wat.edu.pl

**Keywords:** composite materials, plate buckling, fatigue tests, metal plate repair technology, aircraft semi-monocoque structures

## Abstract

Aircraft structures are highly susceptible to fatigue damage, particularly in thin-walled aluminum alloy components such as skin panels. Damage in the form of holes or material loss drastically reduces fatigue life and compromises structural safety, which makes effective repair strategies essential. This study presents an experimental investigation into the fatigue performance of EN AW-2024-T3 aluminum alloy plates with central openings subjected to uniform shear. Repair nodes were applied using two approaches: conventional riveted metal patches and adhesively bonded composite patches. Variants of patch geometry, thickness, and diameter were evaluated to determine their influence on load transfer, buckling response, and fatigue life. The results show that central holes significantly shorten fatigue life, with a 20 mm hole causing a 67% reduction and a 50 mm hole causing a 95% reduction when compared with undamaged plates. Riveted metal patches restored only part of the lost performance, as stress concentrators introduced by fastener holes initiated new fatigue cracks. In contrast, adhesively bonded composite patches provided a substantial improvement, extending fatigue life beyond that of the riveted solutions, improving buckling shape, and delaying crack initiation. Larger patches, particularly those combined with metallic inserts, proved most effective in restoring structural functionality. The findings confirm the effectiveness of bonded composite repairs as a lightweight and reliable method for extending fatigue life and enhancing the safety of damaged aircraft structures. The study highlights the importance of patch geometry and stiffness in the design of repair nodes.

## 1. Introduction

Semi-monocoque structures have been used for many years in aircraft construction and are made mainly of aluminum, magnesium and titanium alloys [1,2]. The primary elements of this type of structure are the frames and skin, which carry the loads applied to the airframe. The aircraft structure is subjected to a complex state of stress due to loading from mass forces, aerodynamic forces and additional loads caused by in-flight overloads or operations such as aircraft takeoff and landing [3]. Emergency damage is difficult to predict, and it typically results from abnormal impacts on equipment (for which it is not designed). Such damage can be caused by collisions of aircraft in flight with birds, collisions on the ground during taxiing with other aircraft or terrain obstacles, and combat actions. Combat damage is understood as the total or partial destruction of an object, punctures, cracks, dents, deformations, and tearing of material or its assemblies, subassemblies, or parts caused by the impact of combat means [3]. Such conditions inevitably lead to fatigue damage, often manifesting as cracks, holes, or local buckling, which significantly reduce both the strength and service life of the structure [4,5].

The purpose of repairs is to restore the strength properties of the structure in the damaged area and to achieve the desired performance characteristics of the repaired section (e.g., in terms of its fatigue life). There are three critical stages in the repair of an aircraft structure, including repair design, material selection, and repair technology.

The methods and materials used in the repair of metal aircraft structures are constrained due to the requirements specific to aircraft design, requirements that are maintained in order to avoid a significant increase in the weight of the aircraft. In practice, the repair joint must meet the requirement of sufficient strength and stiffness so that, on the one hand, it prevents the spread of secondary damage in the repair area and, on the other hand, does not cause additional damage to adjacent structural elements [4]. There are two main methods of repairing aircraft skins, which differ in terms of the material used for the reinforcement patches and the method of joining them to the repaired element: (i) metal patches mounted with mechanical fasteners and (ii) composite patches adhesively bonded to the skin.

In the case of repairs to metallic aircraft skins, the most common repair method involves the use of mechanical fasteners (rivets) to attach a metal repair patch made of the same material as the damaged structure [5]. It is generally assumed that the strength of the repair joint is equal to the strength of the original joint. It should be noted that riveted joints are more susceptible to deformation than bonded joints due to the deformation associated with the compliant rivet seated in the mounting holes and the smaller contact area.

While riveted repairs are relatively simple to implement, they introduce additional stress concentrators through the necessary fastener holes and rarely restore the original fatigue performance. To overcome these limitations, adhesively bonded composite patches have been increasingly investigated as an alternative repair method. Bonded repairs offer several advantages, including reduced weight, elimination of secondary stress concentrators, improved aerodynamic smoothness, and more effective load transfer over a larger surface area [6]. Recent studies confirm their potential in extending fatigue life and delaying crack propagation when compared with riveted solutions [7,8,9].

Despite their drawbacks, studies on such joints demonstrate many advantages compared with the traditional repair method using mechanical fasteners, such as lower weight, smoother aerodynamic shape, the ability to replicate contour shapes, reduced stress concentration around the repair joint [10,11], and effective load transfer, especially in thin-walled components [12,13,14]. The use of composite materials for repairs has contributed, among other things, to [1,15] reducing stress intensity in regions with fatigue cracks and stiffening the damaged area by lowering fatigue stresses in stress concentrators.

The effectiveness of bonded repairs depends strongly on multiple factors, including surface preparation [16,17,18,19,20,21], adhesive selection [22], patch material [23], and repair geometry [21,24]. Optimization of patch geometry and stiffness has been shown to directly affect stress redistribution and the fatigue response of repaired panels [25,26]. Importantly, an excessively stiff patch may accelerate adhesive failure or localize stresses, whereas optimally designed composite reinforcements—typically with a stiffness that is lower than the parent plate—yield more favorable long-term performance [27]. Recent research has focused on optimizing composite patch repairs through improved understanding of material behavior, joint configurations, and repair strategies.

Yang et al. [28] investigated the failure behavior of CFRP composite joints, demonstrating that the ultimate failure load increases with the lap length until reaching a plateau. This finding highlights the importance of properly selecting the overlap length in single-lap joints to maximize strength without unnecessarily increasing weight or material usage. The study also showed that careful joint design can reduce stress concentrations at the edges and improve the overall durability of repaired structures. The study by Xing et al. [24] examined the impact of composite repair patch shapes on the strength of laminated panels. Through uniaxial tensile tests and digital image correlation (DIC) analysis, the research compares circular and square patches, revealing that circular patches offer higher ultimate strength and better repair quality, making them more effective for structural restoration.

Aabid et al. [29] presented a comprehensive review of experimental and numerical research on the application of bonded composite patches for the repair of cracked metallic structures. Their work systematically analyzed how patch geometry, material type, adhesive thickness, and fiber orientation influence the stress intensity factor (SIF) and overall stress distribution in the repaired region. The review highlighted that the use of boron/epoxy and carbon/epoxy patches can lead to significant reductions in SIF—ranging from 60% to over 85%, depending on the repair configuration—demonstrating the high effectiveness of composite patches in mitigating stress concentration at the crack tip. The authors emphasized that properly designed bonded patches not only slow down crack propagation but also restore a substantial portion of the load-bearing capacity of damaged panels. Aabid et al. concluded that continued development of high-strength, fatigue-resistant adhesives and optimization of composite lay-up design could further improve the long-term durability and reliability of bonded repairs.

Baker [14,30,31] extensively investigated the use of composite repair joints, particularly boron–epoxy and carbon–epoxy, for structural repairs. His studies highlighted that adhesively bonding these patches to damaged surfaces can significantly limit the propagation of fatigue cracks. By transferring and redistributing the load around the damaged area, the composite patches reduce stress concentrations at the crack tip, effectively slowing down crack growth. Furthermore, Baker emphasized that the choice of patch material and adhesive, as well as the patch thickness and bonding configuration, play critical roles in achieving optimal repair performance. Pradhan [32] carried out experimental and numerical studies on cracked aluminum plates repaired with boron–epoxy and carbon–epoxy composite patches. The results showed significant reductions in stress intensity factor—up to 54% for double-sided repairs—along with notable increases in load-bearing capacity and fatigue life. The study emphasized the importance of optimizing patch thickness and adhesive properties to achieve efficient stress transfer. Overall, the repaired specimens regained more than 80% of their original strength, demonstrating the high effectiveness of composite patches in structural restoration. Aabid et al. [33] focused on optimizing bonded composite patch repairs using finite element modeling and response surface methodology. They demonstrated that patch geometry, tapering profile, and stiffness ratio strongly influence stress intensity factor reduction, with optimized designs achieving up to an 85% improvement. The research provided practical guidelines for designing high-efficiency, lightweight repair configurations for cracked metallic structures. Their optimized repair models achieved smoother stress distributions and delayed crack propagation, confirming the predictive accuracy of the proposed design approach. Albedah et al. [27] conducted experimental and numerical analyses comparing composite and metallic patches for repairing cracked aluminum alloy 7075-T6 aircraft panels. Their results showed that composite patches—especially boron–epoxy—produced a much greater reduction in the stress intensity factor and delayed crack propagation compared with aluminum patches. The repaired specimens with composite patches exhibited a notable increase in fatigue life, often reaching several times that of the metallic patch repairs, while also maintaining a lower weight and better load transfer efficiency. Finite element simulations confirmed these findings, showing more uniform stress distribution and reduced interfacial stresses in composite-repaired configurations, highlighting their suitability for high-performance aerospace repairs. Moulgada et al. [34] conducted an experimental investigation on cracked plates repaired with various composite patch materials and configurations. It was found that multi-layered patches generally provided superior performance due to increased stiffness and enhanced load transfer, while patch material selection significantly influenced the residual strength and crack-arresting capability. The research underscores the importance of combining material selection, patch geometry, and layering to optimize the effectiveness of composite repairs.

Recent advances in surface treatment techniques for composite-to-metal bonding have demonstrated significant improvements in joint durability under operational environments [35]. Research on the maximum load capacity of joints has also been reported. Kim et al. [36] showed that maximum joint strength occurs when cohesive adhesive failure and delamination of composite adherends happen simultaneously. To further examine the effect of delamination on joint strength, research by Her et al. [37] confirmed that high stress concentrations occur at both ends of the bonding region in adhesively bonded joints, which severely affect the joint’s performance. Similarly, Aimmanee et al. [38] observed that stress concentration in the adhesive layer is still present with locally non-uniform distribution and is always highest at the edges of the bonding region.

Despite these advances, most studies have focused on repairing cracks rather than material loss such as centrally removed sections. In such cases, the repair node must not only restore load transfer across the damaged area but also mitigate local buckling and fatigue crack initiation. This study addresses this gap by experimentally and numerically investigating the fatigue life of EN AW-2024-T3 plates with circular cutouts repaired using two approaches: classical riveted aluminum patches and adhesively bonded glass–fiber composite patches. The influence of patch geometry, thickness, and diameter on fatigue behavior is analyzed. The results contribute to the definition of design guidelines for repair nodes in metallic aircraft structures, highlighting the importance of composite reinforcement as a lightweight and reliable repair strategy.

## 2. Research Methodology

### 2.1. Specimens

**Figure 1:**
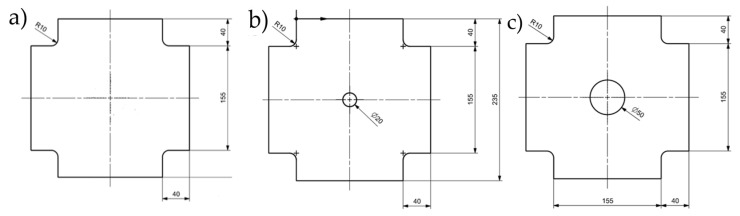
Types of prepared plates: (**a**) undamaged, (**b**) with a 20 mm diameter hole, and (**c**) with a 50 mm diameter hole (unit: mm).

The specimens used for experimental testing were in the form of plates. They were made of 1 mm thick sheet metal (EN AW-2024 T3 aluminum alloy (Batz + Burgel GmbH & Co. KG, Friedberg, Germany)—see Table 1) with dimensions of 240 × 240 mm, and with corner cutouts as shown in Figure 1a. Three variants of the plates were prepared (see Figure 1): (a) undamaged, (b) with a 20 mm diameter hole, and (c) with a 50 mm diameter hole. The holes were used to imitate the occurring damage.

The boundary conditions specified in this case are intended to reflect the nature of the load on the section of the aircraft covering, which is located in the frame between the force elements, i.e., the spars and ribs (in the wing). Due to the limited size of the samples, and so that they could be placed in the strength testing machine, a small area of the surface was analyzed in the form of plates placed in an auxiliary device. Based on the literature [39], a frame-shaped holder was designed and manufactured to hold the samples, which were then placed in the strength testing machine. 

**Figure 2:**
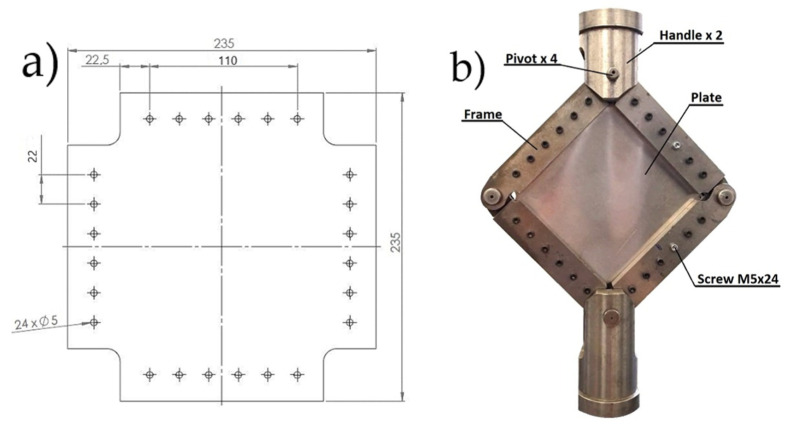
Diagram of (**a**) the placement of mounting holes for plates to be fixed in the frame, and (**b**) the mounting frame with the plate fixed in place (unit: mm).

The frame consisted of 8 steel elements, which, after mounting the specimen, were connected at its edges by means of 14 mm diameter pins. A total of 24 M5 class 12.9 bolts (bolt material yield strength: 1080 MPa) were used to mount the specimen in the frame. The 5 mm diameter mounting holes (six per edge of the plate) were evenly spaced 22.5 mm from the plate edge, with a pitch of 22 mm. The mounting screws were tightened to a torque of 15 Nm. The load diagram of the specimens with the auxiliary device is shown in Figure 2. An MTS 809 axial/torsional test system (MTS Systems Corporation, Eden Prairie, MN, USA) was used for the tests.

### 2.2. Determination of Critical Force

The frame with the plates were placed in an MTS 809 Axial/Torsional Test System testing machine (MTS Systems Corporation, Eden Prairie, MN, USA) and subjected to static tensile loading at a speed of 2 mm/min. The tested specimens, through the test frame, were subjected to shear. The plate under consideration, with all four edges fixed, was subjected to buckling by uniform shear.

A thin-walled plate structure may be subject to buckling, and it is important to determine the critical load that causes it. This is based on the general assumption that an aircraft structure can only be subject to deformation within the range of its elastic deformation. After exceeding the critical load (*P_kr_*), the samples first lost stability, and, when loaded in the post-critical range, they subsequently underwent plastic deformations (permanent buckling). It was assumed that the tested plates, similar to an aircraft skin, are thin-walled components that transfer only shear stresses.

**Figure 3:**
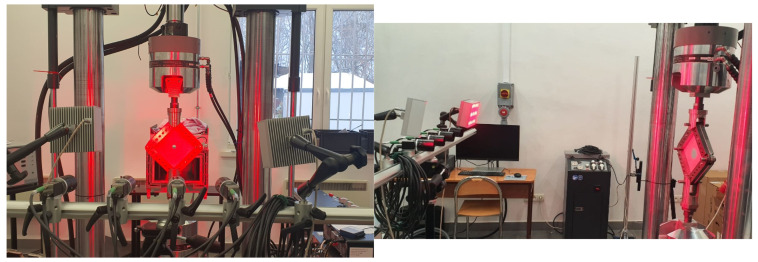
Samples placed in a testing machine during measurement with the DIC system.

Based on the above theory related to the definition of stress in plates subjected to shear, it was decided to determine the critical load and the load causing permanent deformation during the shearing of the tested plates. For this purpose, three undamaged specimens were prepared, onto which speckle patterns were applied. These were used for non-contact strain measurements using the Dantec Dynamics DIC Q-450 digital image correlation system (Dantec Dynamic, Skovlunde, Denmark), which includes four 5 MP cameras with a focal length of 50 mm and a maximum aperture of 1.4, along with the Istra 4D V4.10x64 software system (Dantec Dynamic, Skovlunde, Denmark) (see Figure 3).

### 2.3. Preparation of Plates with Repair Nodes

Restoring the potential of a component to regain its functional properties, such as fatigue life, requires performing a repair. Two repair methods were used in the study: (1) the classic method, using mechanically mounted metal patches, and (2) the composite adhesive method, using patches made of glass composite and attached to the surface with adhesive.

For fatigue tests, plates were prepared with centrally located holes of ø20 mm and ø50 mm, on the basis of which repair joints were made using mechanical connections (rivets and metal patches made from aluminum alloy EA AW-2024-T3) and adhesive joints (adhesive bonds and composite patches made of glass–epoxy composite).

#### 2.3.1. Classic Method

This method involves material, strength, and stiffness similarity—that is, using the same materials from which the damaged elements were made, with geometric dimensions (including thickness) similar to the damaged section of the skin. The strength criterion also implies the need to design a joint between the damaged section and the repair patch that ensures load transfer equivalent to that of the undamaged element.

In order to evaluate the results of the proposed repair solutions, plates with metal repair nodes were made as a reference point. For each variant, three plates with a repair joint were prepared, in which the patches were made of aluminum alloy EN AW-2024-T3 with a thickness of 1 mm, and joined to the repaired structure with rivets. The variants of the applied patch geometries are presented in Table 2. The plates were subjected to harmonic loading cycles in the range of 2–30 kN at a frequency of 2 Hz.

**Figure 4:**
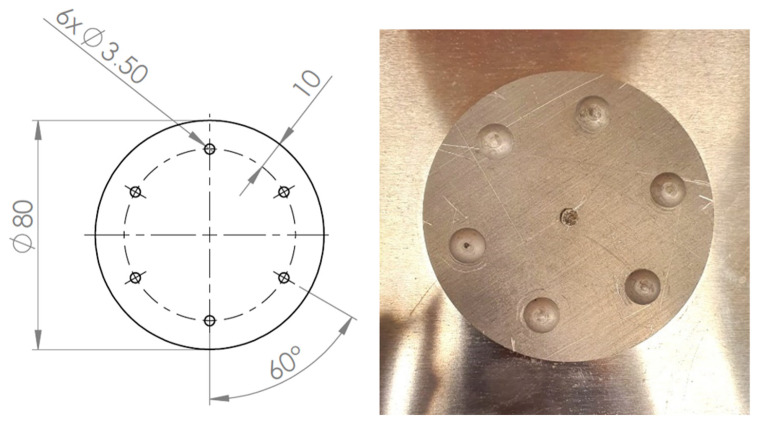
Rivet joints geometry—variant Id (unit: mm).

**Figure 5:**
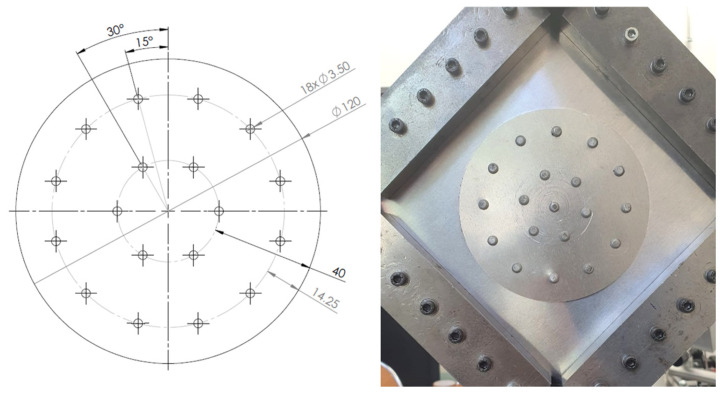
Rivet joints geometry—variant Ie (unit: mm).

**Figure 6:**
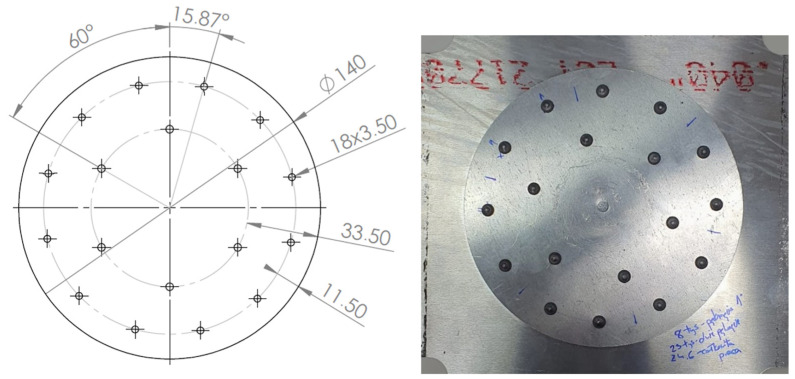
Rivet joints geometry—variant IIc (unit: mm).

The geometries of the rivet joints of variants Id, Ie and IIc are shown in Figure 4, Figure 5 and Figure 6.

#### 2.3.2. Adhesive Method

**Figure 7:**
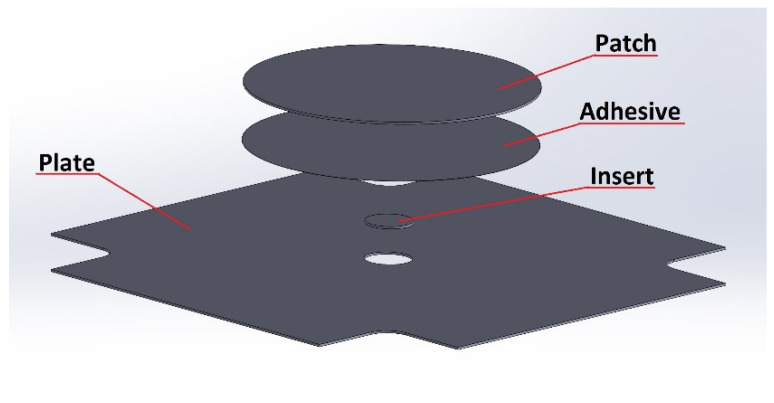
Repair node diagram.

In the case of adhesively bonded repair joints, the repair was performed in the form of a composite patch, a metal insert, and an adhesive layer (Figure 7).

Plates with dimensions shown in Figure 1 were prepared for testing. These included holes with a diameter of 20.50 mm and an insert with a diameter of 20.00 mm (for the Ø20 plate), as well as a hole with a diameter of 50.50 mm and an insert with a diameter of 50.00 mm (for the Ø50 plate). The repair joint of the plate consisted of several components, including a metal insert, a composite patch, and an adhesive layer.

The composite patches were prepared using the hand lay-up method by laminating successive layers of Aeroglass glass fabric with a surface weight of 110 g/m^2^. The glass fabric was impregnated with an epoxy matrix prepared from L285 resin and H285 hardener (MGS, Dortmund, Germany), mixed in a weight ratio of 100:40. The patch was cured under vacuum bag conditions for 24 h at room temperature, and then post-cured at 80 °C for 6 h in a HeatEvent 100/150 thermal chamber (Weiss Technik GMBH, Reiskirchen, Germany).

The diameters of the patches were determined based on the theoretical overlap length (1) [40,41]:

(1) $$l>\frac{5}{m}=\frac{5}{\sqrt{\frac{G_{k}}{\delta_{k}}\frac{\delta_{1}E_{1}+\delta_{2}E_{2}}{\delta_{1}E_{1}\delta_{2}E_{2}}}}\;\;\;$$

where E_1_, E_2_—Young’s modulus of the bonded elements; δ_1_, δ_2_—thicknesses of the bonded elements; δ_k_—thickness of the adhesive layer; G_k_—shear modulus of the adhesive; and l—length of the adhesive joint.

However, given that the repair applies to aircraft structures and that the adhesive joint significantly determines both the strength and, most importantly, the durability of the repair joint, it was assumed that the minimum overlap length should be increased by a multiple.

The prepared composite patches had the diameters listed in Table 3 and consisted of the following:

1. 10 layers arranged according to the lay-up sequence [0°; 45°; 90°; −45°; −90°]_2_, with a thickness of 1 mm (the same as the plate);
2. 16 layers arranged according to the lay-up sequence [0°; 45°; 90°; −45°]_4_, with a thickness of 1.6 mm.

To increase the stiffness of the plate in the damaged area, a metal insert made from the same material as the plate (aluminum alloy EN AW-2024 T3) was inserted into the hole. To facilitate the assembly of the two components of the repair joint—namely, the composite patch and the metal insert—they were joined using a rivet with a diameter of ø1.5 mm, made from PA 25 aluminum alloy (3.9–4.5% Cu, 0.15–0.3% Mg, 0.3–0.5% Mn). The resulting repair patch was then mounted in the damaged area using an adhesive joint.

The plate surfaces in the bonding area were prepared for adhesive bonding in several stages. After initial cleaning with extraction benzine, the surfaces were abraded with 80-grit abrasive cloth (3M, Maplewood, MN, USA), and then cleaned again with extraction benzine after sanding. Before applying the adhesive, a surface treatment agent—Surface Pre-Treatment AC-130-2 Kit (3M, USA)—was applied to the bonding surfaces in accordance with the product’s technical data sheet.

After surface preparation, the adhesive DP420 (3M, USA) was applied to both the repair patch and the plate, and the patch was bonded to the plate under a pressure of 0.8 atm for 24 h at room temperature. To ensure a consistent adhesive layer thickness between the bonded components, 0.1 mm thick spacer threads were introduced. Ref. [42] has confirmed that the use of sol-gel coatings increases surface energy, which can contribute to improved adhesion.

**Figure 8:**
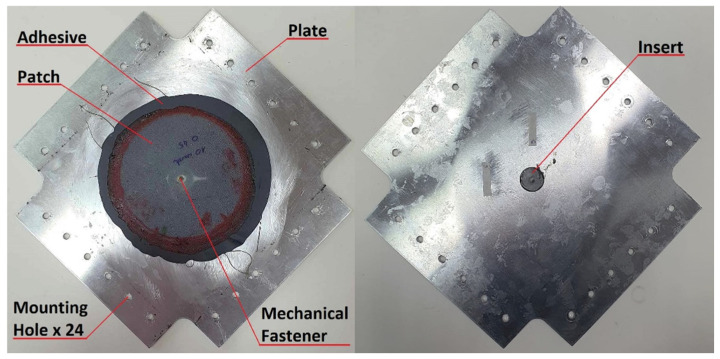
Plate with repair node and mounting holes.

The prepared plate sample with a 20 mm diameter hole and repair joint is shown in Figure 8.

A list of the tested plates is presented in Table 4.

### 2.4. Fatigue Tests

The most important tests for determining the practical applicability of the proposed solutions are fatigue tests, which were conducted to identify the locations where the first fatigue cracks would initiate. Fatigue testing was carried out on undamaged plates, plates with ø20 mm and ø50 mm holes (Figure 1), and repaired plates (using adhesive bonding and mechanical fasteners), using an MTS 809 axial testing machine (MTS Systems Corporation, Eden Prairie, MN, USA). The fatigue tests were performed under a load range of 2–30 kN with a frequency of 2 Hz. The upper load limit was set close to the critical force value of the undamaged plate. This is known as low-cycle fatigue, which occurs when the stress in the samples is higher than 50% of the stress values that cause permanent loss of stability. Such tests are performed in order to shorten the duration of the tests. During the tests, the room temperature was 21 °C and the humidity was around 50% RH.

It is worth noting that the prepared samples were used to conduct a global analysis of the fatigue strength of the proposed element and of how damage affects the fracture location and its durability. In this case, the focus was not on fracture mechanics analysis; it was assumed that the measure of sample weakening/damage was the change in the global stiffness of the entire system and its fatigue life. The stiffness of the system was measured using sensors placed in the crossbar of the strength testing machine; in the event of an increase in its displacement, cracks in the samples were assumed to occur.

## 3. Results and Discussion

### 3.1. Critical Force and Permanent Deformation

**Figure 9:**
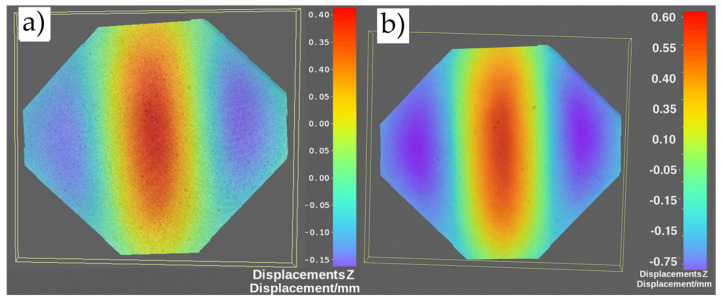
Map of displacements perpendicular to the plate surface under load: (**a**) 11 kN, (**b**) 31 kN.

To determine the critical load value of the undamaged plate, an analysis of images recorded by the Dantec DIC system was carried out, including displacements perpendicular to the sample’s surface (Figure 9a). The onset of wave formation (critical force) in the plate subjected to shear was identified as 11 kN. The load causing permanent deformation of the plate was also determined by analyzing the plate’s out-of-plane displacements under a given load (Figure 9b). In cases where residual deformations were observed after unloading the plate, the applied force was assumed to be the load causing permanent deformation—31 kN. 

Under operational load conditions, local loss of stability of airframe skin panels may occur. In terms of material strength, this phenomenon is equivalent to plate buckling. The load at which buckling occurs is defined as the critical force, and it separates the loading response of the plate into two ranges: subcritical and supercritical. A plate subjected to in-plane loads in the supercritical range may still experience stresses below the yield strength of the material it is made from. This means that, after unloading the aircraft structure, no permanent deformations are recorded in its structural elements.

The present research conducted on plates made of aluminum alloy EA 2024-T3 indicates that the load range up to which no permanent deformations are recorded is relatively wide and may reach almost three times the critical force (range from 11 kN to 31 kN).

### 3.2. Preparation of a Numerical Model

In order to analyze the stress concentration of the tested plates subjected to a load of 30 kN, numerical analyses were performed in Ansys Workbench 2024R1 in the static structure module and eigenvalue buckling in terms of the elastic loss of plate stability. The main objective was to determine the locations of stress concentrations where fatigue cracks may develop and to determine the maximum stress values in the tested plates.

For the purposes of the simulation, a model was prepared in which, in addition to the plate, the plate mounting frame was also modeled, with geometry analogous to that used in the experimental tests (see Figure 2). The material properties of the plate were assumed to be those of an AW-2024 T3 series aluminum alloy, and the values of the multilinear material model are presented in Table 5.

The model takes into account the mounting of the plate in a steel frame using 24 M5 steel screws and 4 steel pins. For the purposes of the simulation, the material parameters of the frame elements, bolts, and pins were assumed to be as follows: Young’s modulus 220 GPa and Poisson’s ratio 0.3 [-]. The material data were imported from the engineering data program library.

The model mesh consisted of 97,716 Tet10 and Hex20 elements and 179,078 nodes. In terms of boundary conditions, contacts between individual model elements were defined as follows: plate frame of the frictional type with a coefficient of 0.1, and frame pins and frame bolts of the no separation type.

**Figure 10:**
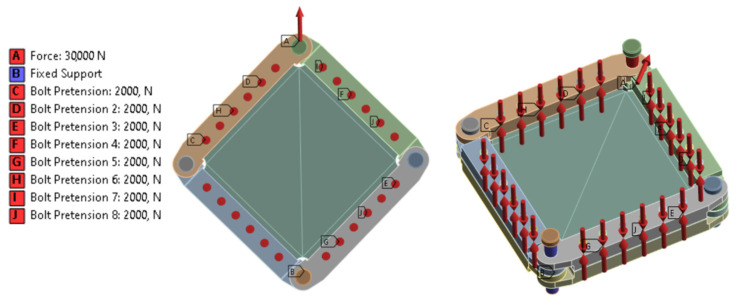
Defined boundary conditions in the model in the form of force, restraint, and bolt tension.

In terms of load conditions, a force load with a value of 30 kN was defined for pin (A), while for pin (B), the following displacement and rotation conditions were defined using the Fix Support function: Fx;Fy;Fz = 0; Mx;My;Mz = 0. For the mounting bolts, a Bolt PreTension type pre-assembly load with a value of 2000 N was defined for a single bolt (Figure 10).

**Figure 11:**
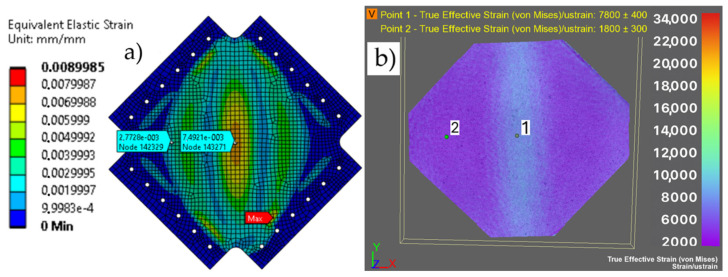
Von Mises reduced strain for a load of 30 kN (**a**) in the numerical model [mm/mm] and (**b**) measured by DIC [µm/m].

The obtained von Mises stress map for a plate loaded with 30 kN was verified with von Mises stress values obtained from measurements using DIC instrumentation at comparable points (Figure 11 and Table 6).

It can be assumed that the model is consistent with the actual object and that the obtained deformation values are similar to each other, and it was assumed that the numerical model can be used for further analysis of the plate stress.

**Figure 12:**
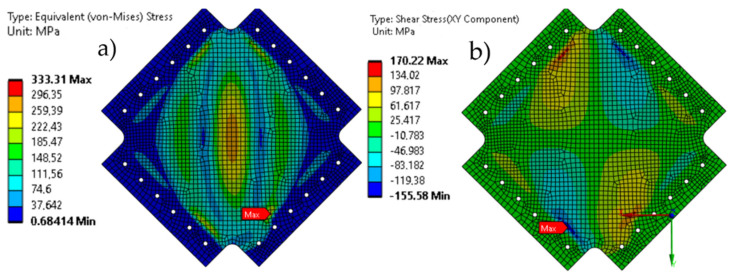
(**a**) von Mises stress map of the plate and (**b**) XY shear stress map of the plate.

The maximum von Mises stresses occurring in the plate loaded with 30 kN have a value of 333 MPa, which is the maximum permissible value in the range of elastic deformations (330 MPa). These occur at the center point of the plate and at the edges of the mounting frame (Figure 12a). Based on the shear stress map on the XY surface of the plate, local areas of stress concentration were observed near the mounting frame and in areas of geometry changes, confirming the influence of geometric discontinuities on the stress state (Figure 12b). In the analyses performed, no stress concentrations were observed directly in the mounting holes.

### 3.3. Fatigue Tests—Results

When analyzing the results of fatigue tests, sample variances and standard deviations for a 95% confidence interval were calculated for each variant considered.

#### 3.3.1. Plate Without a Hole

**Figure 13:**
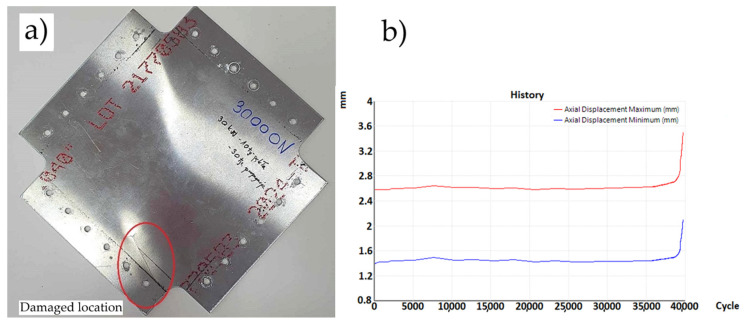
(**a**) Sample without a hole—crack propagating from the attachment point—and (**b**) graph of displacement as a function of cycles (sample without a hole) loaded with a cycle of 30—2 kN.

For the case of a plate without a hole, three fatigue tests were performed. At 35,900, 34,500 and 33,400 load cycles, fatigue cracks initiating and propagating from the mounting holes of the samples appeared (Figure 13a). The sample variance is 1,570,000 cycles^2^ and the standard deviation is 1253 cycles. The fatigue test graph (Figure 13b) shows the constant value of the maximum and minimum displacements of the plate, as well as the moment when the crack occurred and its propagation, causing an increase in the displacement values. It was observed that the graph showing the displacement of the crossbar from the number of cycles at the moment of crack formation shows slight vibrations. Based on this observation, it was assumed that this is the moment when cracks form, which are not always visible because they are sometimes located under the mounting holders. 

#### 3.3.2. Plates with a 20 mm Diameter Hole

**Figure 14:**
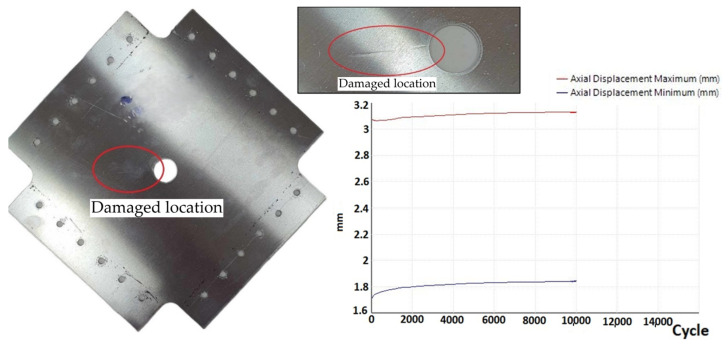
Sample with a 20 mm diameter hole—crack at the hole.

In three plates with a 20 mm diameter hole, fatigue cracks appeared after 10,800, 9200 and 12,700 cycles, with crack propagation always starting from the hole (Figure 14). The sample variance is 3,070,000 cycles^2^ and the standard deviation is 1751 cycles. Crack propagation occurred perpendicular to the direction of the load on the sample.

#### 3.3.3. Variant Ia

**Figure 15:**
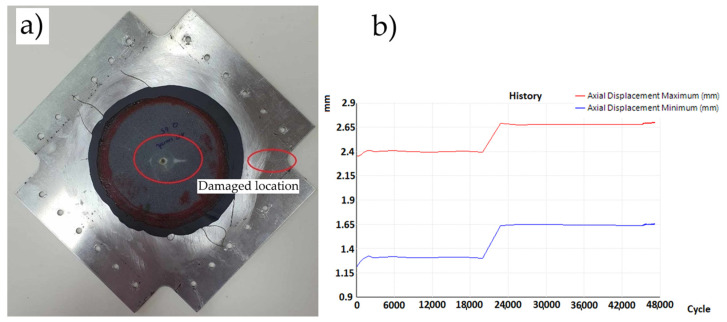
(**a**) Option I—crack at the hole and edge—and (**b**) displacement graph as a function of the number of cycles for the ø20 sample with a 1 mm hole.

In three plates repaired using variant Ia, fatigue damage appeared after 17,500, 22,500, and 20,000 cycles at the side edges of the sample, in the area where the structure was under tension. The sample variance is 6,250,000 cycles^2^ and the standard deviation is 2500 cycles. After approximately 30,000 ± 3500 cycles, a crack appeared near the hole and became visible through the repair patch (Figure 15a). As the fatigue cracks propagated, a linear degradation of the adhesive bond between the plate and the repair patch was also observed. The recorded displacement changes during the test are presented in Figure 15b. The visible change in strain indicates the initiation and propagation of a crack at the edge of the sample.

#### 3.3.4. Variant Ib

**Figure 16:**
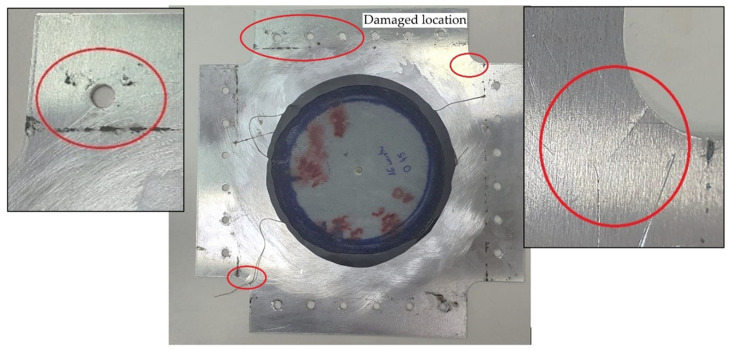
Variant Ib—crack on the edge, crack in the mounting holes.

After 72,700, 70,200, and 74,900 cycles, a crack was observed on one edge, while, after another 23,900 ± 2000 cycles, a crack appeared on the other edge (Figure 16). The sample variance is 5,530,000 cycles^2^ and the standard deviation is 2352.66 cycles. The cracks propagated from the edges perpendicular to the applied load. Cracks originating from the mounting holes were also observed after removing the plate from the mounting frame.

The plate exhibited more than three times the fatigue life compared with the plate in variant Ia, twice the fatigue life of the undamaged plate, and over six times that of the plate with a hole. The damage mode was similar to that observed in the variant Ia sample.

#### 3.3.5. Variant Ic

**Figure 17:**
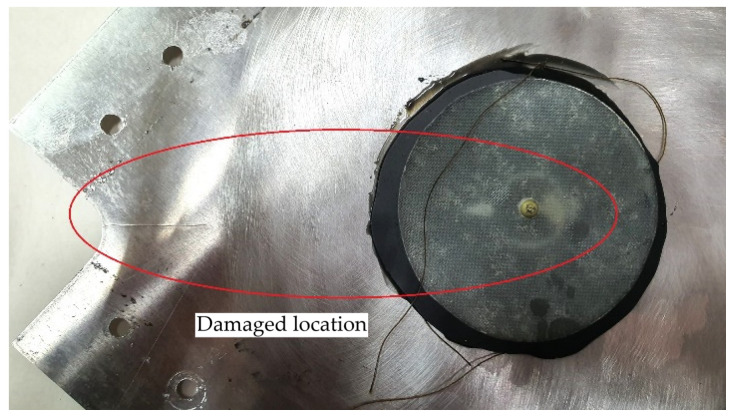
Variant Ic—crack at the edge and near the hole in the plate, detachment of the patch near the hole.

After 74,100, 72,300, and 70,200 cycles, a crack was observed at the edge of each of the three plates, and after an additional 6000 ± 1000 cycles, another crack appeared near the hole (Figure 17). The sample variance is 3,810,000 cycles^2^ and the standard deviation is 1952.56 cycles. During fatigue tests, a similar wave-like deformation pattern was observed as in the case of the plate without a hole.

#### 3.3.6. Variant Id

**Figure 18:**
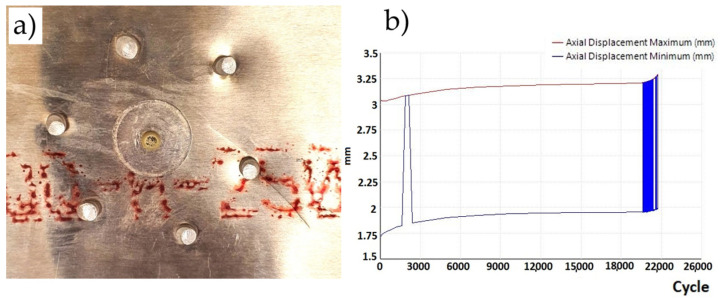
Variant Id—(**a**) crack at the rivet joints with deformation of the material at the mounting holes and (**b**) displacement graph as a function of the number of cycles.

After 19,700, 18,400, and 20,900 cycles, a crack was observed at the rivet joints (Figure 18a). The sample variance is 1,562,667 cycles^2^ and the standard deviation is 1250.07 cycles. Figure 18b shows an increase in the displacement of the machine crossbeam. During each of the three fatigue tests, it was observed that patches did not deform together with the plate. Deformations of the material can be observed at the mechanical fasteners.

#### 3.3.7. Variant Ie

**Figure 19:**
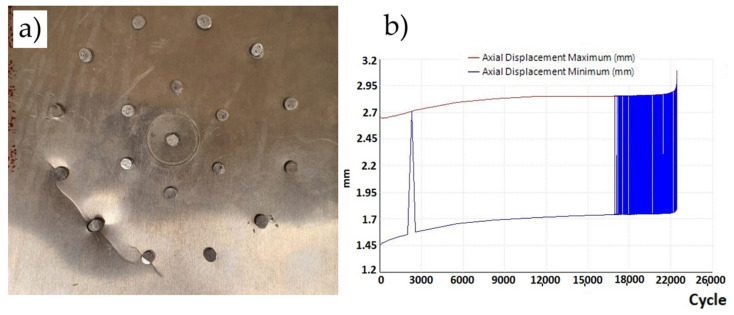
Variant Ie—(**a**) rupture from rivet joints and (**b**) displacement graph as a function of the number of cycles.

After approximately 19,700, 20,400, and 18,900 cycles, a crack was observed at the rivet joints (Figure 19). The sample variance is 563,317 cycles^2^ and the standard deviation is 750.54 cycles. During the fatigue test, it was observed that the patch deformed together with the plate.

Table 7 presents the arithmetic mean of the fatigue test results obtained for the plates.

All of the repair variants increased the fatigue life of the damaged specimens. Fatigue life greater than that of the undamaged sample was achieved using thicker patches—16-layer patches—of 1.6 mm (variants Ib and Ic). In the case of variants Id and Ie with riveted patches, the original fatigue life was not restored, and the intervention in the repaired material introduced additional notches, which weakened the structure. Regardless of the number of rivets used or the diameter of the metal patch, the fatigue life of these plates exhibited similar values.

The size of the composite patch in the repair node was defined by the overlap length of the adhesive bond between the damaged plate and the composite patch. In the case of variant Ic, where a minimum overlap length was assumed—and which was determined analytically from the formula (1)—the patch diameter for a 20 mm hole was selected as 70 mm. Although asymmetric deformation shapes were observed during fatigue testing, the repaired plate showed a higher fatigue life than the plate with a hole.

After applying a larger patch (122 mm diameter), the buckling shape resembled that of an undamaged plate. In both repair variants Ib and Ic—representing smaller and larger patch diameters—with identical patch thickness, the fatigue life was comparable. However, in the case of the larger patch, no cracks occurred in the damaged area of the plate. The authors of [44] emphasize that, when selecting the parameters of the patch and the bonded joint, not only should its strength, stiffness, and geometric dimensions be taken into account, but also the type of deformation caused by the use of a given solution, which may affect the manner of the destruction of the repair joints and their strength.

An important component of the repair node is the insert filling the hole in the plate. Its presence ensures a single-wave buckling shape with a more favorable curvature at the peak. Furthermore, the initiation of fatigue cracks around the hole occurs later, indicating a positive influence of the insert on the fatigue life of the repair. As the insert was made from the same material as the plate and plays a significant role in the repair, it was mechanically connected to the patch using a rivet.

#### 3.3.8. Plates with a ø50 mm Diameter Hole

**Figure 20:**
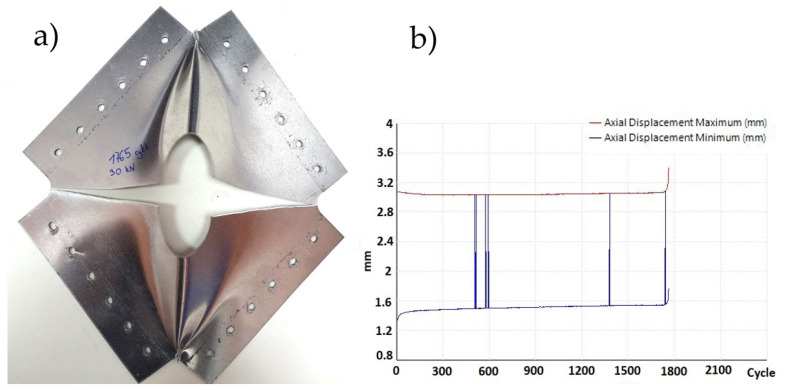
(**a**) Sample with a 50 mm diameter hole—plate rupture—and (**b**) graph of displacement as a function of cycles (50 mm diameter) loaded with a cycle of 30—2 kN.

In three plates with a 50 mm diameter hole, after 2090, 1420, and 1780 cycles, the plates always cracked along the axis of the hole perpendicular to the direction of the load on the sample (Figure 20a), in each case without any previously visible cracks (Figure 20b). The sample variance is 112,449.56 cycles^2^ and the standard deviation is 335.31 cycles. 

#### 3.3.9. Variant IIa

**Figure 21:**
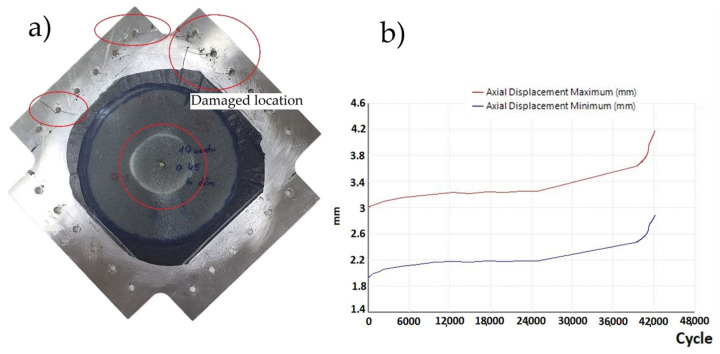
(**a**) Variant IIa—crack in mounting holes—and (**b**) displacement graph as a function of number of cycles.

In the plates repaired in variant IIa, fatigue damage appeared after 23,200, 24,900, and 26,800 cycles at the mounting location between the plate and the frame (in the mounting holes). The sample variance is 3,242,500 cycles^2^ and the standard deviation is 1801 cycles. Additionally, under loading, the reinforcing insert detached from the plate and partially detached from the patch (Figure 21a). The recorded displacement changes during the test are shown in Figure 21b. The noticeable increase in strain indicates the initiation and propagation of cracks beneath the mounting fixture.

#### 3.3.10. Variant IIb

**Figure 22:**
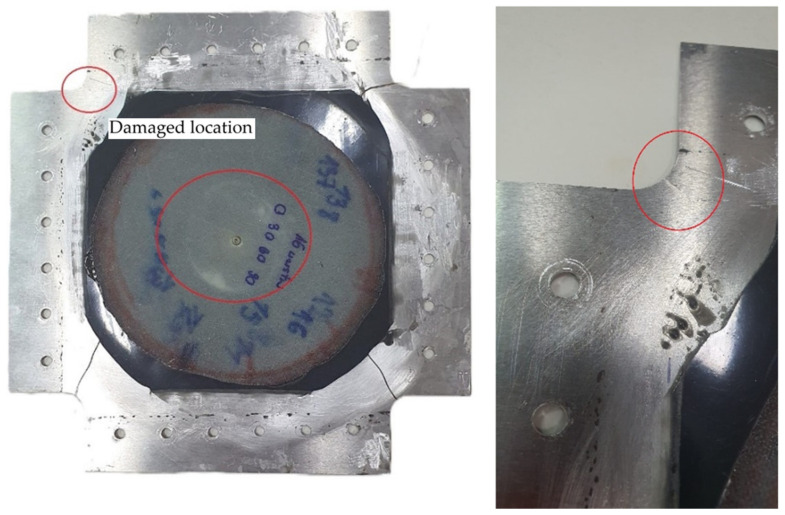
Variant IIb—crack at the edge.

In the plates repaired in variant IIb, fatigue damage appeared after 64,800, 66,900, and 62,100 cycles at the edge of the plate (Figure 22). The sample variance is 5,790,000 cycles^2^ and the standard deviation is 5406.25 cycles. In addition, when the plate was loaded, the reinforcement insert detached from the plate and partially detached from the patch.

#### 3.3.11. Variant IIc

**Figure 23:**
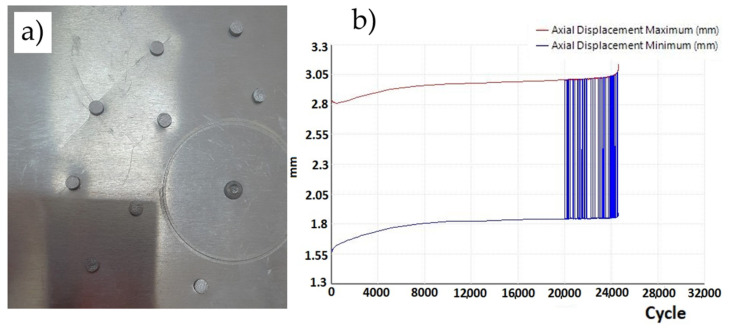
(**a**) Variant IIc—crack at mounting holes and (**b**) displacement graph as a function of number of cycles.

In each of the three repaired plates in variant IIc, fatigue damage appeared after 7400, 8100, and 8700 cycles, originating from the mounting holes (Figure 23a). The sample variance is 423,333.33 cycles^2^ and the standard deviation is 650.64 cycles.

The recorded displacement changes during the test are shown in Figure 23b. At approximately 8000 cycles, the plate exhibited strain hardening, and the displacement of the machine’s crosshead remained constant.

Table 8 presents the arithmetic mean of the fatigue test results obtained for the plates.

In the case of undamaged plates and plates with 20 mm and 50 mm holes, different crack locations and mechanisms were observed. For undamaged plates, cracking occurred at the mounting holes. In contrast, for plates with holes, cracking initiated at the edges of the cut holes due to stress concentrations and material discontinuities. These are expected cracking mechanisms and are consistent with observations in aircraft structures.

The aim of the research was to highlight the reduction in fatigue strength of the overall structure caused by different levels of damage (undamaged, and plates with 20 mm and 50 mm holes). In the case of the tested plates, damage in the form of a centrally placed hole led to the following reductions in fatigue life:

- For a 20 mm diameter hole (which accounted for 4% of the plate area), fatigue life was significantly reduced by 67% (from 34,500 thousand to 11,500 cycles).
- For a 50 mm diameter hole (10% of the plate area), fatigue life was reduced by 95% (from 34,500 to 1765 cycles).

Restoring the functional properties of the element (e.g., fatigue life) requires repair. After classical repair, recovering the original performance was a challenge, especially in terms of fatigue life. A key limitation in fatigue life restoration results from the need to drill mounting holes for rivets, which act as stress concentrators and initiate fatigue cracks.

When mechanical fasteners were used to attach a metal patch made of the same alloy as the damaged plate (thickness 1 mm), the following fatigue life improvements were observed:

- For plates with a 20 mm hole, an increase of 70% (from 11,500 to 19,600 cycles), regardless of the patch diameter or number of rivet rows.
- For plates with a 50 mm hole, an increase of 353% (from 1765 to 8000 cycles).

Other researchers have come to similar conclusions [44], observing that the use of thicker composite patches bonded to the surface resulted in up to a threefold increase in the fatigue life of the tested specimens compared with those that were damaged. Other research [45] has revealed results in which composite patches increase the fatigue life of cracked structures significantly, ranging from 55% to 100% for different applied stresses.

In classical solutions, the stiffness of the patch—measured as the product of its thickness and Young’s modulus—is chosen to match the stiffness of the repaired plate. Based on the research using composite patches with adhesive joints (which, unlike mechanical joints, are surface bonds rather than point bonds), it was found that the patch stiffness should be lower than that of the repaired plate.

When using adhesive bonds, it is also important to keep in mind the significant influence of the surface preparation process on the strength parameters of this type of bond and its fatigue life [46].

## 4. Conclusions

(1) Effect of damage:
  - Holes in thin-walled aluminum alloy plates (EN AW-2024-T3) drastically reduce fatigue life.
  - A 20 mm hole reduced fatigue life by ~67%, while a 50 mm hole reduced it by ~95%.
(2) Effectiveness of repair methods:
  - Classical riveted metal patches restore only part of the lost fatigue life and introduce additional stress concentrators from fastener holes.
  - Adhesively bonded composite patches are more effective, providing longer fatigue life, better buckling behavior, and delayed fatigue crack initiation.
(3) Influence of patch geometry and size:
  - Patch diameter: Larger patch diameters improved stress distribution around the damaged zone, resulting in reduced local stress concentrations and more uniform load transfer.
  - Fatigue crack initiation: Smaller patches (e.g., 70 mm) extended fatigue life but still allowed crack initiation near the hole; larger patches (e.g., 120–150 mm) delayed or eliminated crack formation within the repaired area.
  - Buckling shape: Larger patches restored a buckling pattern closer to that of the undamaged plate, leading to more stable deformation under load.
  - Patch thickness: Thicker composite patches (1.6 mm, 16 layers) provided significantly higher fatigue life compared with thinner patches (1 mm, 10 layers).
  - Patch stiffness ratio: Optimal results were obtained when the patch stiffness was lower than the plate stiffness (~30% of the base plate). Patches that were too stiff led to stress localization and premature adhesive failure.
(4) Role of repair node inserts:
  - Metallic inserts in repair nodes improved buckling shape, provided more favorable load distribution, and delayed crack initiation.
  - Inserts combined with composite patches enhanced overall repair performance.
(5) General findings:
  - Adhesively bonded composite repairs are a lightweight and effective method for restoring strength and extending fatigue life in damaged aircraft structures.
  - Proper surface preparation, optimal patch geometry, and balanced stiffness are essential to repair quality.

## 5. Future Recommendations

In order to broaden the scope of application of these results, the impact of the use of patch diameters on the fatigue life of plates and their stiffness should be examined in more detail. In addition, the impact of temperature, humidity, and UV exposure on the strength parameters of composite repair joints is an important issue that should be considered in further research on this topic. Future work will help to generalize the current conclusions for use in adhesive repairs of aircraft in various metal structures subjected to different phenomena.

## Figures and Tables

**Table 1:** Mechanical properties and chemical composition of EN AW-2024 T3 aluminum alloy (Germany).

Modulus of Elasticity [GPa]	Young’s Modulus [MPa]	Poisson’s Ratio	Density [g/cm^3^]	Alloying Elements
73.1	330	0.33	2.78	Aluminum (90.7–94.7%)
				Copper (3.8–4.9%)
				Magnesium (1.2–1.8%)
				Manganese (0.3–0.9%)
				Silicon (0.5%)
				Iron (0.5%)
				Zinc (0.25%)
				Titanium (0.15%)
				Chromium (0.1%)

**Table 2:** Variants of metal patch geometry and number of mechanical fasteners.

Variant No.	Patch Thickness [mm]	Rivets Diameter [mm]	Number of Rivets	Patch Diameter [mm]
ø20 mm				
Id	1	3.5	6	80
Ie			18	120
ø50 mm				
IIc	1	3.5	18	140

**Table 3:** Geometric parameters of the assumed diameters of the patches.

**Plate with ø20 Hole**	
Patch diameter	70 mm
	120 mm
**Plate with ø50 Hole**	
Patch diameter	120 mm
	150 mm

**Table 4:** Variants of manufactured plates with repair nodes.

Variant No.	Connection	Patch Thickness [mm]	Patch Diameter [mm]
ø20 mm			
Ia	Adhesive	1	120
Ib		1.6	120
Ic		1.6	70
Id	Mechanical	1	70
Ie		1	120
ø50 mm			
IIa	Adhesive	1	150
IIb		1.6	150
IIc	Mechanical	1	140

**Table 5:** Strength values of AW-2024 T3 aluminum alloy [43].

Material Name	Young’s Modulus [GPa]	Poisson’s Ratio [-]	Deformations [-]	Stresses [MPa]
AW-2024 T3	70	0.36	0	330
			0.0098	348.45
			0.0196	370
			0.0385	410.8
			0.0741	468.72
			0.1071	507.36
			0.1379	540.56

**Table 6:** Summary of von Mises stresses for both measurement cases—FEM and DIC.

Type	Von Mises Strain [mm/mm]	
	Point 1	Point 2
FEM model	7.47 × 10^−3^	2.77 × 10^−3^
DIC	7.80 × 10^−3^	1.80 × 10^−3^

**Table 7:** Arithmetic mean of fatigue test results for maximum cycle force of 30 kN—ø20 mm plates.

Specimen Type	Cycle No.	Confidence Interval [Cycles]	Lower and Upper Limits of the Confidence Interval [Cycles]
Without hole	34,600	±3113	(31,487–37,713)
With ø20 mm hole	10,900	±4350	(6550–15,250)
Ia	20,000	±2500	(13,791–26,209)
Ib	72,600	±5848	(66,752–78,448)
Ic	72,200	±4853	(67,347–77,053)
Id	19,667	±3129	(16,538–22,796)
Ie	19,667	±2115	(17,552–21,782)

**Table 8:** Arithmetic mean of fatigue test results for a maximum cycle force of 30 kN—ø50 mm plates.

Sample Type	Number of Cycles	Confidence Interval [Cycles]	Lower and Upper Limits of the Confidence Interval [Cycles]
Without hole	34,500	±3113	(31,487–37,713)
With ø50 mm hole	1763	±832	(931–2595)
IIa	24,967	±4482	(20,485–29,449)
IIb	64,600	±5974	(58,626–70,574)
IIc	8067	±1616	(5451–9983)

## Data Availability

The original contributions presented in this study are included in the article. Further inquiries can be directed to the corresponding author.
